# Pathology of Diphtheria

**Published:** 1889-03

**Authors:** F. S. Johnson

**Affiliations:** Professor of General Pathology and Pathological Anatomy in the Chicago Medical College, Chicago, Illinois


### PATHOLOGY OF DIPHTHERIA.

BY F. S. JOHNSON, M. D.,

Professor of General Pathology and Pathological Anatomy in the Chicago Medical College, Chicago, Illinois.

Diphtheria is an acute, specific, infectious and contagious disease, with local and general manifestations and characteristics.

The local lesions are found in the upper air passages, especially the pharynx, but also in the nose, larynx, trachea and deeper parts of the respiratory tract. These may, however, occur primarily in other parts of the body. The general manifestations are fever and concomitant symptoms.

The local manifestations are inflammation and the formation of a false membrane on the surface, or in the superficial portions of the tissue.

From the clinical standpoint it is difficult to say whether diphtheria is primarily a general or local disease. The general symptoms usually appear before the local trouble is manifested. On the other hand, the local trouble sometimes exist with scarcely noticeable general symptoms.

The poison seems to usually gain access to the body with the inspired air.

By analogy the pathogenesis of the disease is at the present time commonly attributed to micro-organisms, although no form has yet been found to satisfy the conditions formulated by Koch, and held by all experimenters as necessary for proof. Micro-organisms are sometimes found in the kidneys, but according to Birch-Hirschfeld only in cases of severe sepsis.

1. The micro-organism must be present in all cases of the disease.

2. Pure cultures of this micro-organism must be capable of producing the disease.

3. The micro-organism must be found in the tisse of the animal in which the disease is experimentally produced.

No one form has yet been found to be invariably present. No form has been found which produces the characteristic phenomena of the disease, and which does not give rise to other forms of poisoning. In experiments upon animals the results of inoculation have, in most instances, either been negative or more nearly resembling the phenomena of pyæmia or septicæmia. All the forms in diphtheritic membranes have been found in the human mouth under normal conditions.

The recent researches into the mode of action of micro-organisms in disease, have led to a change in the generally accepted views upon the subject. It is now held that micro-organisms do not in themselves act as poisons, or to any extent mechanically by forming capillary emboli, but that by the splitting up of proteid substances, they produce ptomaines and set free ferments, and that these are agents in the causation of disease.

Anatomically classified the local phenomena of diphtheria are:

a, catarrhal inflammation; b, croupous inflammation; c, diphtheritic inflammation.

Topographically classified the disease is diphtheria when it occurs in the pharynx and nose; croupous or laryngeal diphtheria when it occurs in the larynx.

It is proper here to mention other specific disease-forms with similar or identical local manifestations : Diphtheritic inflammations occurring in the course of the exanthematous fevers ; wound diphtheria ; dysentery ; puerperal endometritis ; diphtheritic cystitis.

Ætiologically considered, there may be as many forms as there are agents capable of destroying tissue and exciting inflammation.

In extremely mild cases the local changes are indistinguishable from those of simple catarrhal inflammation, and anatomically they belong to this class. The damage to the mucous membrane by the specific poison, is here not sufficient to destroy any part of it, but simply to excite a moderate inflammation. And under these conditions the exudation from the blood vessels, in reaching the surface, is filtered through the epithelium of the mucous membrane and is thereby rendered non-coagulable, and removed from the parts affec ed in the manner as are the normal secretions. In these cases there is no false membrane, though the cause is the specific poison of diphtheria. To what extent these mild cases occur it is impossible to say, because the presence or absence of a false membrane usually determines the diagnosis.

The false membrane or diphtheritic membrane is the result of combined inflammation, tissue death, and coagulation within the densely infiltrated tissue. The thickness of the membrane varies with the depth to which the tissue is destroyed. The essential anatomical distinction between simple catarrh and diphtheritic forms of inflammation of mucous membranes, lies in death of the tissue, especially of the epithelium. When the epithelium has been removed, in whatever way, inflammatory exudation upon the denuded surface will coagulate, thus forming a false membrane. The destruction of the epithelium by chemical agents and heat is followed by the formation of a coagulum upon the surface, or, in other words, a false membrane.

Anatomically, there are two varieties of pseudo-membranes, croupous and the diphtheritic. In the former the epithelium only is destroyed and the depth of the pseudo-membrane is limited by the basement membrane of the mucosa. In the latter the underlying tissues also suffer death. Mucosa, submucosa, and even the deeper tissues are involved in the formation of the pseudo-membrane.

In the pathological processes the ætiological agent,—namely, the diphtheritic poison, is the prime factor. It seriously impairs the vitality of the tissues or destroys them outright. This injury produces inflammation and the already damaged tissues are infiltrated and swollen by the copious exudation from the blood vessels. Death of the entire mass soon follows, and with it coagulation. This coagulation of tissue and inflammatory exudate constitutes the false membrane. The greater the injury to the tissue, the deeper the processes reach and the more intense the inflammation. The character of the exudation depends upon this condition. In the milder forms the exudate are made up almost wholly of leucocytes and plasma; in the severer forms, large numbers of red blood corpuscles are extravasated and the infiltration is more or less hæmorrhagic in character.

The diphtheritic membrane usually appears first upon the tonsils, sometimes upon the soft palate, or even in the nose; and not infrequently in the larynx. At first it is a thin, irregularly oval white or grayish patch, or even merely a lace-like layer of fibrinous shreds upon the surface. When fairly formed, the membrane is tough and firmly adherent.

In croupous membranes the firmness of the attachment depends in a measure upon the character of the underlying mucous membrane. When this is closely adherent to the parts beneath, the pseudo-membrane is less easily cast off. Thus in the larynx pseudo-membranes are more firmly attached to the vocal cords and to the lower surface of the epiglottis than over other parts. As the local changes go on, the tissue becomes involved to a greater depth; and this extreme infiltration adds to the bulk of coagulable material. Thus, what began as a croupous membrane, may become diphtheritic in character. As it undergoes degeneration, it becomes yellowish and fatty. It is gradually cast off as a whole, or in shreds or layers.

In severer cases the coagulum contains a variable proportion of red blood corpuscles, and the membrane is darker and greenish.

In the most severe forms the affection spreads rapidly from the beginning; the membrane is dirty, hæmorrhagic in appearance, and foul-smelling.

Gangrene seldom occurs, and when present it is probably not due to the intensity of inflammatory processes, but to the direct effect of the virulent poison. The false membrane may sometimes, in all probability, be the seat of invasion by micro-organisms capable of causing pyæmia or septicæmia. In these cases the individuals may suffer from this infection in addition to the existing diphtheria, in the same manner as though infection through any exposed wound. This condition may seriously complicate the symptoms of the primary disease. Gangrene, too, may be due to other causes than the diphtheritic poison act ing upon the already damaged tissues.

Histologically, pseudo-membranes consist of homogeneous flakes and strands and fibrinous threads enmeshing leucocytes and red blood corpuscles. Frequently the quantity of leucocytes is so great as to obscure the fibrinous coagulum. Remnants of the former tissues are also included in the membrane. As the membrane undergoes degeneration, the fibrin becomes finely granular and the appearance of a network is lost. The tissues immediately underlying the false membrane are densely infiltrated. On tearing off the latter the surface is mottled red and pale; there are seen bleeding points, interstitial hæmorrhages, and shreds and masses of adherent and more or less degenerated pseudo-membrane.

The membrane may extend from the primary seat to surrounding parts, as from the tonsils to other parts of the pharynx, into the eustachian tubes, and the nose, seldom into the æsophagus; more frequently into the larynx, trachea and bronchi. When stenosis of the larynx occurs, there is varying acute emphysema ot the upper lobes and hyperæmia of the lower lobes of the lungs. If, when in this condition, the smaller bronchii are invaded, or are closed by aspiration of bits of pseudo-membrane, there is occlusion of the bronchi and broncheoles and consequent atelectasis, and lobular pneumonia. These conditions rarely occur in the upper lobes, because of the emphysema and consequent anæmia. Under these circumstances, if air is freely admitted to the lungs by tracheotomy, the upper lobes are more readily infected.

With general infection of the body are associated more or less extensive pathological changes in the various tissues and organs. Some of these are manifested chiefly by derangement of physiological functions.

The anatomical changes result from the direct effect of the noxa upon the tissues.

The most constantly observed secondary local infection in pharyngeal diphtheria is that of the lymphatics behind the jaw and the superficial glands of the neck. These are often highly inflamed and much swollen.

In laryngeal diphtheria the swelling of the superficial glands is usually absent, since the lymphatics of the larynx are more deeply situated and swelling in them often escapes notice.

Hæmorrhages are common in the various organs and from serous surfaces. The heart muscle and voluntary muscles undergo fatty changes; waxy degeneration has also been observed.

In the kidneys, even in the lighter cases, there is cloudy swelling of the epithelium. In severer cases there is frequently glomento-nephritis, hæmorrhage, extensive degeneration of the epithelium in the convoluted tubes, and sometimes diffuse nephritis. The spleen is hyperæmic, the malphigian bodies often enlarged, as are the lymphatics of the body generally. In the mucous membrane of the alimentary canal the lymph follicles are often swollen, sometimes there is superficial ulceration. In the liver there is a more or less degree of fatty degeneration. Diphtheritic inflammation of the stomach seldom occurs, and then is confined to the cardiac portion. In the brain and cord menngeal and parenchymatous, hæmorrhages sometimes occur.

Klebs has found accumulations of micro-organisms in the perivascular lymph spaces of the brain, and Dejerine has found accumulation of white and red blood corpuscles similarly situated in the spinal cord.

In three cases of extensive diphtheritic paralysis he found advanced degeneration of the nerve fibres of the anterior root, and degeneration of ganglion cells in the anterior horns. In most of the cases of paralysis, the disturbance is referable to degenerative changes in the peripheral nerves.

